# Estimation of the Iron Loss in Deep-Sea Permanent Magnet Motors considering Seawater Compressive Stress

**DOI:** 10.1155/2014/265816

**Published:** 2014-08-06

**Authors:** Yongxiang Xu, Yanyu Wei, Jibin Zou, Jianjun Li, Wenjuan Qi, Yong Li

**Affiliations:** Department of Electrical Engineering, Harbin Institute of Technology, West Dazhi Street, Harbin 150001, China

## Abstract

Deep-sea permanent magnet motor equipped with fluid compensated pressure-tolerant system is compressed by the high pressure fluid both outside and inside. The induced stress distribution in stator core is significantly different from that in land type motor. Its effect on the magnetic properties of stator core is important for deep-sea motor designers but seldom reported. In this paper, the stress distribution in stator core, regarding the seawater compressive stress, is calculated by 2D finite element method (FEM). The effect of compressive stress on magnetic properties of electrical steel sheet, that is, permeability, BH curves, and BW curves, is also measured. Then, based on the measured magnetic properties and calculated stress distribution, the stator iron loss is estimated by stress-electromagnetics-coupling FEM. At last the estimation is verified by experiment. Both the calculated and measured results show that stator iron loss increases obviously with the seawater compressive stress.

## 1. Introduction

In recent ocean exploitation, permanent magnet motors have been increasingly applied owing to the benefits of high efficiency and low maintenance costs [1–4]. For deep-sea applications, the ambient pressure exerted by seawater can reach a very high level that makes pressure-proof protection indispensable. Compared with pressure hulls, fluid compensated pressure-tolerant system dispenses with thick case and is more favorable for low weight and low cost applications [5, 6].

**Figure 1:**
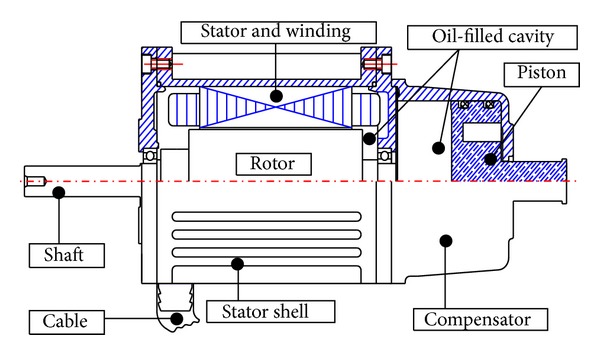
Deep-sea PMSM with oil-filled compensator.

Generally, the realization of pressure-tolerant systems is done with fluid compensated and casted systems. A deep-sea PMSM with oil-filled compensator is shown in Figure 1. When the motor submerges deeper, the piston shifts inward to balance the increased seawater pressure. Then the stator will be compressed by both seawater outside and oil within the motor. The balanced pressure inside and outside the motor imposes uniform compressive stress on the whole stator core. In addition, since both the outer and inner surfaces of the stator core are compressed, the stress direction is varied due to the tooth-slot structure.

As has been confirmed by experiment results, the compressive stress acts on the electrical steel sheet can deteriorate the magnetic properties and increase the iron loss [7–10]. Therefore the estimation of iron loss in deep-sea permanent magnet motors must take into account the seawater compressive stress.

Since the theoretical model of the increase in iron loss caused by stress is far from mature at present [11], researchers always pursue approximate model by virtue of empirical methods. The common estimation procedure involves 3 subtopics: determination of stress distribution, magnetic properties measurement of stator core under compressive stress, and iron loss calculation based on the former topics.

Determination of stress distribution is frequently realized by finite element method (FEM), while experimental method can also be found in reports. In this paper FEM is applied. For conventional land type motors, compressive stress in stator core is induced mostly by the production process. Shrink fitting and stamping are identified as the main sources. Generally, the stress distribution is not uniform in the whole stator core but confined in some specific regions. For example, the effect of press-fitting only acts upon the yoke and the teeth are almost not affected. Obviously, it is quite different from the stress induced by deep-sea environment which acts on the whole stator. In fact different stress distributions can change the magnetic properties of stator core in different way.

The measurement of magnetic properties of stator core under compressive stress has been extensively reported in literature for land type motors. To impose stress on the specimen, various exclusive apparatus are presented varying from purpose-built mechanical rig [7] to hydraulic equipment [9, 12, 13]. However, stress is always exerted in a single direction by mechanical load to imitate the stress distribution in land type motor. Therefore, a novel stress exerting method is needed to imitate the polydirectional stress in deep-sea motor. In this paper, a pressure tank is applied to produce a similar high pressure environment as deep sea.

As for iron loss calculation, it is dominated by stress-electromagnetics-coupled 2D FEM at present. The effect of shrink fitting, punching, and anisotropy of nonoriented core on iron loss of actual motor is examined using measured properties of stator core in [14]. The iron loss of IPMSMs driven by PWM inverters is calculated using the combination of stress analysis and electromagnetic field analysis that considers the transverse stress effect and motor deformation [15]. However, there is hardly any paper reported addressing the influence of seawater compressive stress on the iron loss of deep-sea permanent magnet motors.

Since the effect of stamping can be avoided by preparing stator core using wire cutting [16], in this study only shrink fitting is considered. In this paper, stress distribution is studied taking both shrink fitting and seawater pressure into account. Then the degradation in the magnetic properties of the stator core is measured with certain compressive stress. Based on these, an estimation of the iron loss using mechanical stress-electromagnetics-coupled 2D FEM is presented. The effect of compressive stress on stator iron loss, as well as the estimation result, is at last verified by experiment.

## 2. Stress Distribution in the Stator Core considering Seawater Compressive Stress

**Figure 2:**
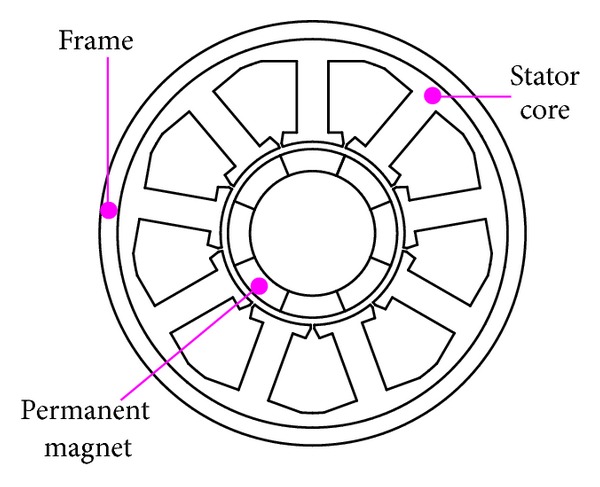
2D FEM model of studied motor (coils are not shown).

The 8-pole 9-slot surface permanent magnet motor (SPM) model investigated in this paper is shown in Figure 2. Primary specifications are listed in Table 1. The stator core is stacked with nonoriented electrical steel sheet (grade: WTG1500) and the axial length is 120 mm. The ring frame is made of titanium alloy to withstand high pressure in the deep-sea environment. The stator back core and the frame are fixed together by shrink fitting. For simplicity, the surface rib of ring frame is ignored in this model.

In 2D plane, components of stress are defined as

(1) $$\begin{array}{c} \sigma_{x}=\frac{E}{\left(1+\nu \right)\left(1-2\nu \right)}\left\{\left(1-\nu \right)\varepsilon_{x}+\nu \varepsilon_{y}\right\}\\ \sigma_{y}=\frac{E}{\left(1+\nu \right)\left(1-2\nu \right)}\left\{\left(1-\nu \right)\varepsilon_{y}+\nu \varepsilon_{x}\right\}, \end{array}$$

where *E* is Young's module, *ν* is Poisson's ratio, and *ε* is the strain caused by both the shrink fitting and the compressive stress.

The compressive stress in the stator core is always evaluated using the Von Mises stress. The Von Mises stress is given by the following equation:

(2) $$\begin{array}{c} \sigma_{v}=\sqrt{\sigma_{x}^{2}-\sigma_{x}\sigma_{y}+\sigma_{y}^{2}}. \end{array}$$

The stress caused by shrink fitting is usually analyzed using the heat strain induced from the thermal difference during the fitting process [10]. In this paper, the thermal difference between the stator and the frame is set as 65°C according to the actual case. It should be noted that shrink fitting only compresses the outer surface of the stator core.

When the motor operates on land, the normal atmospheric environment exerts a 0.1 MPa pressure. When it submerges to about 7000 m deep sea, the compressive stress of seawater is 70 MPa. The hydraulic pressure acts on both the outside surface of ring frame and the inner surface of stator core and the direction is perpendicular to the surface. Therefore, in the FEM model the effect of circumstance pressure takes the form as an equivalent compressive stress in perpendicular direction setting on the outside surface of frame and inner surface of stator core.

**Figure 3:**
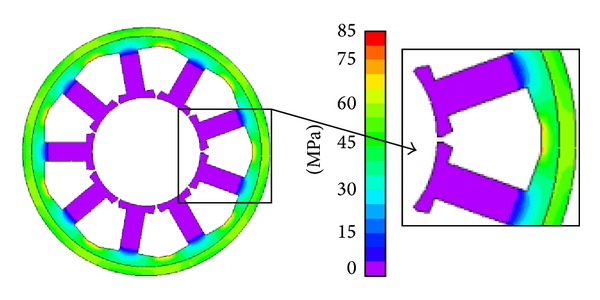
Von Mises stress distribution in stator core on land.

**Figure 4:**
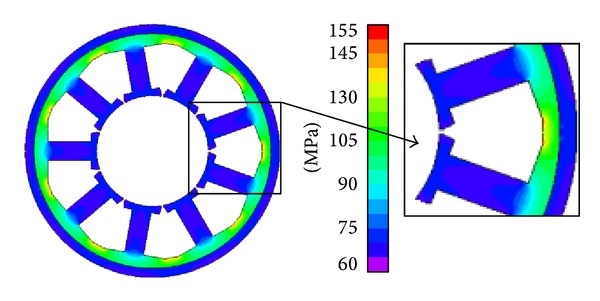
Von Mises stress distribution in stator under 70 MPa stress.

Based on the conditions above, the stress distribution in the stator core caused by shrink fitting and ambient compressive stress is calculated using commercial FEM software. Figures 3 and 4 show the results. It can be seen that considerable difference in stress distribution arises when the motor, respectively, operates on land and under 7000 m deep sea.

In Figure 3, the surface pressure, both outside and inside of the motor, is 0.1 MPa. Large stress can be seen in the back yoke and the ring frame while the stress distributed in the stator teeth is almost zero. That means the effect of shrink fitting does not act upon the whole stator core but is confined in the yoke. The stress in the ring frame is almost at the same level with that in the back yoke. The maximum stress (81.4 MPa) arises at the separated part of the stator back yoke.

In Figure 4, the outside and inside pressure of the motor are 70 MPa. Here because both the outer and inner surfaces of the stator core are compressed, the stress distribution spreads into the teeth. Similar to Figure 3, the stress in the back yoke is larger than that in the stator teeth. However, the value in the ring frame is much closer to that in the stator teeth than back yoke. The average stress in teeth is 68 MPa. And the maximum stress in the stator back yoke is 151.8 MPa.

## 3. Influence of Compressive Stress on Magnetic Properties of Electrical Steel Sheet

Due to mechanical stress, the magnetic characteristics of electrical steel become worse and the iron loss increases. To evaluate the effect of seawater compressive stress on the magnetic properties of iron steel, a novel experimental method is proposed in this paper, by which the magnetic properties, that is, permeability and B-H and B-W curves, are tested in this paper.

### 3.1. Measuring System

**Figure 5:**
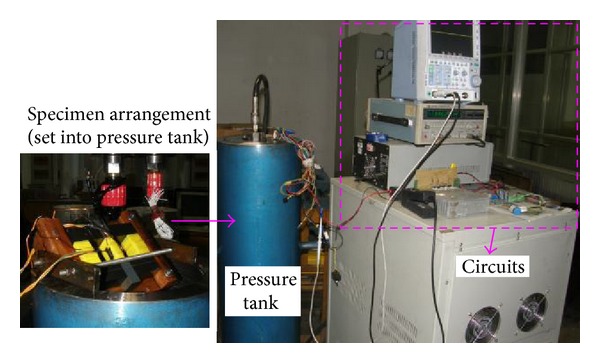
Measuring system.

To simulate the compressive stress applied on the actual stator core, a measuring system is designed in laboratory as shown in Figure 5. The system is composed of a pressure container, a set of exciting and measurement circuits, and a specimen arrangement. The pressure container is actually a pressure-tight tank filled with hydraulic oil. Its end cover is equipped with cable connector by which the external circuits are connected with the specimen coils. The specimen arrangement is put into the tank during the test and, by adjusting the pressure in the tank, the specimen is compressed with different stresses.

**Figure 6:**
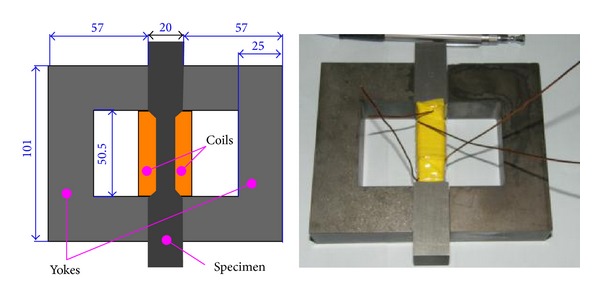
Construction of specimen arrangement and photo.

Figure 6 shows the construction of the specimen arrangement. It is composed of 2 yokes, one laminated specimen, and two coils (exciting coil and measuring coil). The yokes are made of soft magnetic ferrite. The specimen is laminated by 120 pieces of nonoriented electrical steel sheet (grade: WTG1500, the same as the studied motor stator). To avoid the influence of punching on magnetic properties of steel sheets, the specimen is prepared by wire cutting. It is worth noting that this specimen arrangement is substantially a transformer: the exciting coil is primary side, the measuring coil is secondary side, and the specimen and yokes form a closed magnetic circuit. The flux density B in the specimen is proportional to the voltage of the measuring coil while the magnetic field strength H in the specimen can be calculated by detecting the current of the exciting coil. It should be noted that, guaranteed by the close-loop exciting circuit, all the magnetic property measurements are carried out under sinusoidal flux condition [17].

### 3.2. Measuring Results

Magnetic properties of the laminated specimen, that is, the permeability and B-H and B-W curves, are measured under various stresses by applying a 400 Hz sinusoidal flux.

**Figure 7:**
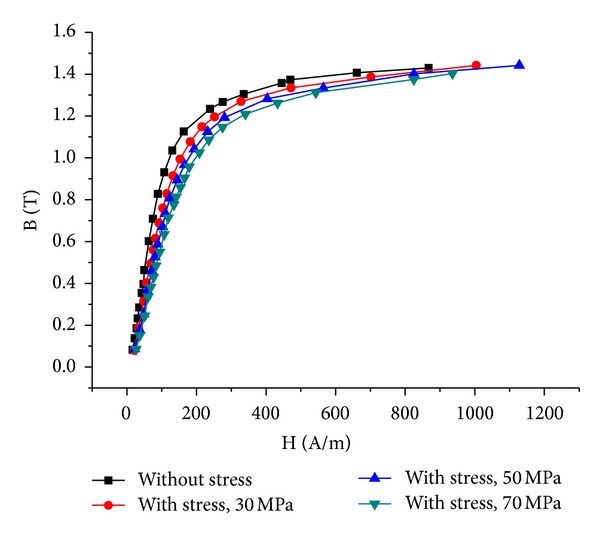
B-H curves under different stresses.

**Figure 8:**
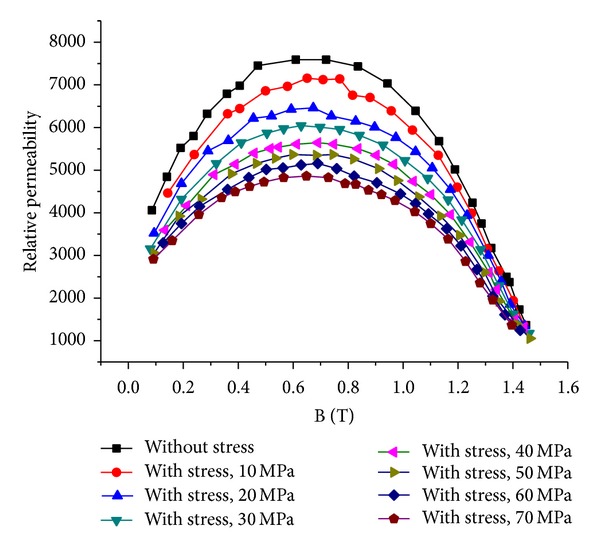
Relative permeability under different stresses.

Figures 7 and 8 show the measured B-H curves and permeability of the steel sheet under different stresses. It can be seen that the permeability decreases rapidly with the increase of compressive stress and the decrease rate becomes smaller gradually.

**Figure 9:**
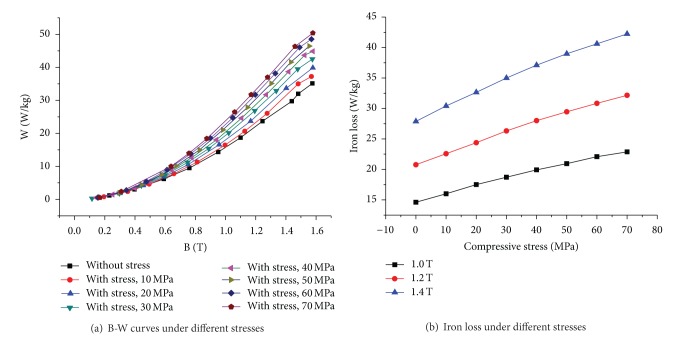
Effect of compressive stress on iron loss of electrical steel.

Figure 9 shows the effect of compressive stress on the iron loss. It is shown that the iron loss increases rapidly along with the increase of compressive stress and the rate of increase becomes smaller gradually. Compared with under normal atmospheric environment, the iron loss increases with a factor of 1.5 times under 70 MPa pressure (at 1.4 T flux density).

## 4. Estimation of Iron Loss

Based on the results of stress distribution calculation and magnetic properties measurement, iron loss of stator core under seawater pressure is evaluated using 2D electromagnetic transient FEM calculation considering no load operation of the motor.

### 4.1. Flux Distribution and Iron Loss Density in Stator Core

**Figure 10:**
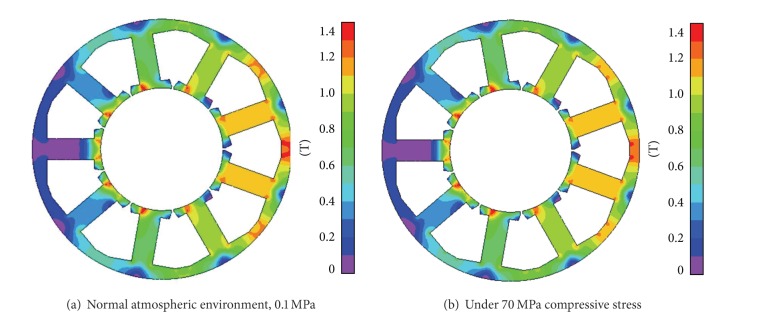
Flux distribution in the stator core.

**Figure 11:**
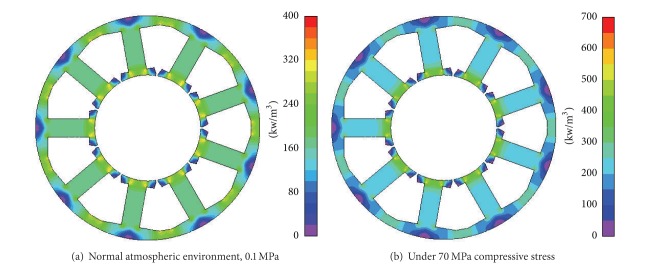
Iron loss density in the stator core.

By setting the speed of motor at 6000 rpm (400 Hz electrical frequency), the flux distribution and iron loss density in stator core are calculated. The results are shown in Figures 10 and 11.

The maximum flux density in Figure 10(a) is 1.42 T while in Figure 10(b) it is 1.39 T. This small difference in flux density is caused by the difference between the compressive stresses as shown in Figures 3 and 4. Although the huge compressive stress considerably degrades the magnetic conductance of the stator core, a sharp decrease of the flux density in stator is avoided thanks to the large equivalent air gap caused by thick permanent magnets.

However, the case is not so for iron loss. In fact a sharp increase of iron loss density in the stator core arises when the compressive stress is imposed. In Figure 11(b), the maximum iron loss density is 686 kW/m^3^, which is much higher than that in Figure 11(a), 382 kW/m^3^.

#### 4.1.1. Stator Iron Loss

**Figure 12:**
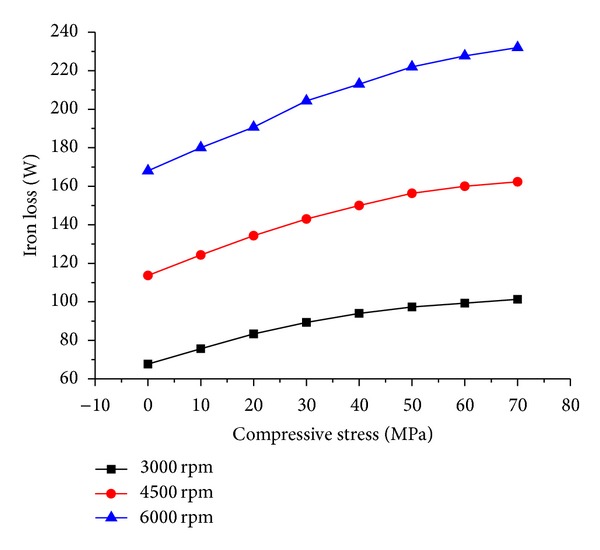
Effect of compressive stress on stator iron loss.

Under certain compressive stress, stator iron losses at various operation speeds are calculated and the results are shown in Figure 12.

It can be seen that the stator iron loss increases obviously with the rise of stress, but the rate of increase reduces as the stress increases. At the speed of 6000 rpm, compared with normal atmosphere the iron loss of motor increases to 1.4 times when the motor is compressed by 70 MPa pressure.

## 5. Experimental Verification

**Figure 13:**
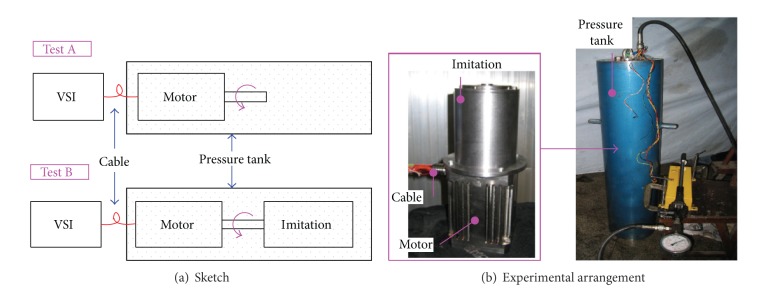
Experiment systems for iron loss measurement.

To measure the effect of ambient compressive stress on stator iron loss, an experiment scheme is designed as shown in Figure 13(a). The actual experimental arrangement is shown in Figure 13(b).

An imitational motor is manufactured according to the studied motor. The imitation is almost a copy of its template, except that its rotor has nonmagnetized permanent magnets. When external voltage source inverter (VSI) drives the motor at the speed of 6000 rpm, the imitation will rotate as an accompaniment on condition of coaxial coupling as shown in Figure 13(a) Test B.

Power loss of the system can be measured by detecting the input power on the cable. Then the power losses of the two tests in Figure 13(a) are as follows [5]:

(3) $$\begin{array}{c} p_{A}=p_{m}+p_{Fe}+p_{o}\\ p_{B}=\left(p_{m}+p_{Fe}+p_{o}\right)+\left(p_{m}+p_{o}\right), \end{array}$$

where *p*_*Fe*_ is the iron loss, *p*_*o*_ is the viscous drag loss, and *p*_*m*_ is the mechanical loss. It should be noted that with nonmagnetized permanent magnets the imitation has no iron loss. Then, subtract *p*_*B*_ from two times *p*_*A*_, and iron loss is separated:

(4) $$\begin{array}{c} p_{Fe}=2p_{A}-p_{B}. \end{array}$$

**Figure 14:**
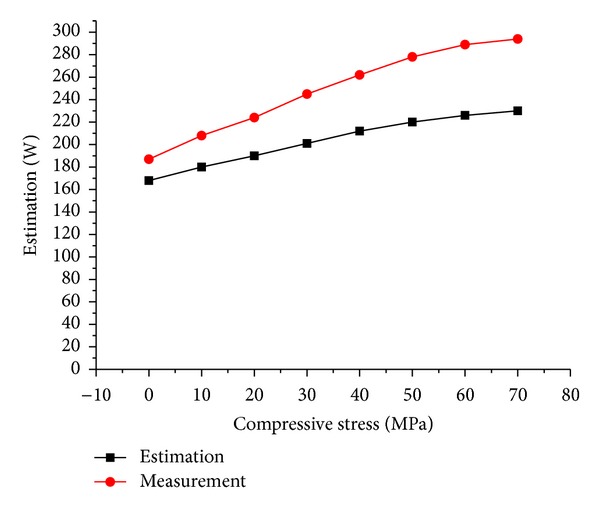
Measured and estimated iron loss under different stresses.

Measured iron losses under different stresses, together with the estimated ones, are shown in Figure 14. It should be noted that both the estimation and experiment are conducted under no load operation of the motor.

It is clear that the two curves show the same trend as the compressive stress increases. In other words, the estimated and measured results are coincident showing that stator iron loss increases obviously with the seawater compressive stress.

In addition, the measured iron loss under 70 MPa pressure is 1.58 times of that under normal atmosphere while the estimated increase factor is 1.4 times.

Although the estimation is verified by the measurement, a considerable divergence exists between them. The possible reason would be that the iron loss of steel sheets shown in Part III is measured with alternating magnetic field while the actual operational magnetic field in motor is rotating field. The iron loss in rotating magnetic field can be much bigger than that in alternating magnetic field [7]. Therefore our further study will deal with this subject.

## 6. Conclusion

The estimation of stator iron loss in a deep-sea PMSM is investigated considering the stress distribution. The results show that

1. the oil inside and seawater outside compress the stator core significantly in both the teeth and the back yoke. The stress distribution in stator core is quite different from land type motors;
2. large compressive stress exerted by seawater deteriorates the magnetic properties of the electrical steel. The permeability of the steel sheet decreases obviously while the iron loss increases rapidly. The increase rate of iron loss reduces gradually along with the climb of compressive stress;
3. compared with on land operation, the stator iron loss of the studied deep-sea PMSM increases 40% under 70 MPa seawater compressive stress at the rated speed;
4. based on the results of stress distribution calculation and magnetic properties measurement, the iron loss of stator core is estimated using stress-electromagnetics-coupling FEM. The estimated result is verified by the measured result.

## Figures and Tables

**Table 1:** Primary specifications of the studied motor.

Dimension	Value	Parameter	Value
Stator outer diameter	106 mm	Dc-link voltage	110 V
Stator inner diameter	50 mm	Rated torque	10 N*·*m
Active axial length	120 mm	Rated speed	6000 rpm
